# CD4/CD8 ratio normalization rates and low ratio as prognostic marker for non-AIDS defining events among long-term virologically suppressed people living with HIV

**DOI:** 10.1186/s12981-018-0200-4

**Published:** 2018-09-27

**Authors:** Win Min Han, Tanakorn Apornpong, Stephen J. Kerr, Akarin Hiransuthikul, Sivaporn Gatechompol, Tanya Do, Kiat Ruxrungtham, Anchalee Avihingsanon

**Affiliations:** 1 HIV-NAT, The Thai Red Cross AIDS Research Center, 104 Ratchadamri Rd., Pathumwan, Bangkok, 10330 Thailand; 2 Faculty of Medicine, Chulalongkorn University, Bangkok, Thailand

**Keywords:** CD4/CD8 ratio, Immune restoration, Non-AIDS events, Long-term virological suppression, Asia

## Abstract

**Background:**

Immune restoration is often incomplete after ART in HIV patients, both quantitatively and qualitatively. We studied the incidence and probability of CD4/CD8 normalization in an adult Thai HIV cohort and explored the predictive value of the ratio for developing of non-AIDS defining events (NAEs).

**Methods:**

We analyzed data from HIV-infected Thai adults between 1996 and 2017 in the HIV-NAT 006 prospective long-term cohort in Bangkok, Thailand. Normalization was defined as CD4/CD8 ratio ≥ 1 on two consecutive visits, and normalization probability was calculated using the Kaplan–Meier method. NAEs were a composite endpoint including cardiovascular or cerebrovascular diseases, chronic kidney diseases, non-AIDS defining malignancies and death. Multivariate Cox regression was used to evaluate demographic, disease and treatment characteristics associated with CD4/CD8 ratio normalization and NAEs.

**Results:**

A total of 800 ART-naïve patients with baseline CD4/CD8 ratio of < 0.8 who started combination ART, and had sustained virological suppression were enrolled. Participants were on ART for a median of 8.9 years and virologically suppressed for 6.1 years. The probabilities of CD4/CD8 normalization at 2, 5 and 10 years after virological suppression were 5.1%, 18.6% and 39.1%, respectively. Factors associated with normalization in multivariate analysis were female sex (hazard ratio [HR]: 2.47, 95% CI 1.71–3.56, p < 0.001) and baseline CD4 counts ≥ 350 cells/mm^3^ (HR: 3.62, 95% CI 2.36–5.55), p < 0.001) vs. < 200 cells/mm^3^ as reference. The second analysis explored the predictive value of CD4/CD8 ratio for NAEs. Older age (HR: 1.09, 95% CI 1.05–1.13, p < 0.01) and current CD4/CD8 ratio < 0.3 (HR: 3.02, 95% CI 1.27–7.21, p = 0.01) or between 0.3 and 0.45 (HR: 2.03, 95% CI 1.03–3.98, p = 0.04) vs. > 0.45 were independently associated with higher risk of progression to NAEs in the multivariate analysis.

**Conclusions:**

Our findings showed that complete immune recovery is uncommon in an Asian setting and earlier ART initiation at higher CD4 counts may have increased the ratio sooner. The findings demonstrate the use of CD4/CD8 ratio as a prognostic marker for clinical progression of NAEs.

*Trial registration* HIV-NAT 006 cohort, clinical trial number: NCT00411983

## Background

Survival of HIV-infected individuals has significantly improved with the introduction of combination antiretroviral therapy (cART). Nowadays, since antiretrovirals (ARVs) are initiated in earlier stages of infection, HIV-associated illnesses or complications of acquired immunodeficiency syndrome (AIDS) are less frequently observed in most HIV-infected individuals [1]. Despite low risks of AIDS in many patients who are treated with cART, immune recovery or restoration is rarely achieved [2, 3]. The incidence of several serious non-AIDS defining events (NAEs) including cardiovascular disease, cerebrovascular disease, renal disease and non-ADIS-related cancers have risen as longevity has increased among PLHIV [4]. Lifestyle factors and ageing play a role, but the impact of HIV infection and associated chronic inflammatory states on the incidence of these NAEs is still not fully understood.

Chronic inflammation includes persistent ongoing immune activation, bacterial translocation caused by injured mucosa-associated lymphoid tissue, asymptomatic replication of HIV itself and co-pathogens such as cytomegalovirus infection. These conditions lead to immune activation, and hence potentially increase the risk for all-cause mortality in HIV patients [5–8]. The ratio of CD4^+^ T cells to CD8^+^ T cells (CD4/CD8 ratio) has been used as a surrogate marker of immunosenescence in both the general population [9, 10] and PLHIV [11]. Additionally, the CD4/CD8 ratio has shown an independent association with NAEs and mortality in people living with HIV (PLHIV) whereas CD4 counts alone do not predict the risk of NAEs [12, 13]. Studies have reported the ratio remains low, with a slow rate of recovery in a substantial proportion of patients, including those with adequate CD4^+^ T cell count recovery, over years of cART [12, 14]. The clinical significance of this ongoing immune dysfunction needs further exploration.

The majority of previous published studies exploring associations with CD4/CD8 ratios and their prognostic value for predicting NAE have been from Western countries, and evidence to inform an association in HIV-infected Asians is still limited. A study comparing Asian and Caucasian cohorts has shown that the baseline CD4/CD8 ratio before cART initiation was significantly lower in Asians but there were no differences in the odds of achieving the ratio value of > 1 between two cohorts [15].

In this study, we aimed to firstly evaluate the incidence and probability of CD4/CD8 normalization and to explore factors associated with normalization among patients from a Thai HIV cohort with sustained virologic suppression and long-term follow-up. Secondly, we explored the value of CD4/CD8 ratio in predicting NAEs including cardiovascular or cerebrovascular diseases, kidney diseases, non-AIDS malignancies and deaths.

## Methods

### Study design and population

This is a prospective study in an ongoing HIV Thai adult cohort (HIV-NAT 006 cohort, clinical trial number: NCT00411983). The details of this cohort have previously been described [16–18]. Participants were eligible for inclusion in this analysis if they achieved and maintained suppressed viremia at HIV-RNA < 50 copies/mL after starting cART. Once a participant had detectable viral load after achieving suppression, follow-up for that participant was censored. All patients had regular clinic visits where medical history, including the occurrence of NAEs was documented.

### Definition of endpoints

The normalization of CD4/CD8 ratio, defined as two consecutive values ratios ≥ 1 [19], was the primary endpoint of the initial analysis. For the second analysis, we defined NAEs as cardiovascular or cerebrovascular diseases (including coronary artery diseases, myocardial ischemia, cerebral artery occlusion and stroke), chronic kidney diseases (defined as confirmed estimated glomerular filtrate rate < 60 mL/min with Modification of Diet in Renal Disease MDRD formula), non-AIDS defining malignancies and deaths (excluding 1 death from heroin overdose, 1 unknown cause of death, 3 suicide cases).

### Statistical analysis

Firstly, the probabilities of CD4/CD8 ratio normalization and associated factors were identified. Baseline for CD4/CD8 normalization analysis was the first of two consecutive virological suppressions after initiating cART. The normalization incidence rate was calculated by dividing the number of normalization events by total person-years of follow-up (PYFU) after achieving viral suppression. The Kaplan–Meier method was used to assess independent predictors associated with a normalized CD4/CD8 ratio. Baseline covariates modelled were age, sex, mode of HIV transmission, HIV-RNA levels, CD4 and CD8 cell counts, Centers for Disease Control and Prevention (CDC) staging, hepatitis B surface antigen (HBsAg), anti-hepatitis-C antibody (anti-HCV ab).

Secondly, the incidence rate of NAEs was analyzed as a composite endpoint by combining deaths and other NAE in time to event models. CD4/CD8 ratio was fitted as categories with cutoff values of < 0.30, 0.30–0.45 and > 0.45 [11, 12], for the interests of applicability in clinical practice. A value of CD4/CD8 ratio < 0.4 showed the best cutoff to predict NAEs [11] and to link the association with different T-cell activation markers [2]. In these models, NAEs or deaths were modelled as a first event; patients not reaching the endpoint were censored at their most recent clinic visit. Time-updated covariates modelled included were age, body mass index (BMI) and tuberculosis infection. Diabetes mellitus was defined by two consecutive values of fasting blood glucose ≥ 126 mg/dL or initiation of anti-diabetic medications. Covariates in univariate analysis with p value < 0.15 were included in the multivariate model.

## Results

### CD4/CD8 ratio normalization

**Fig. 1:**
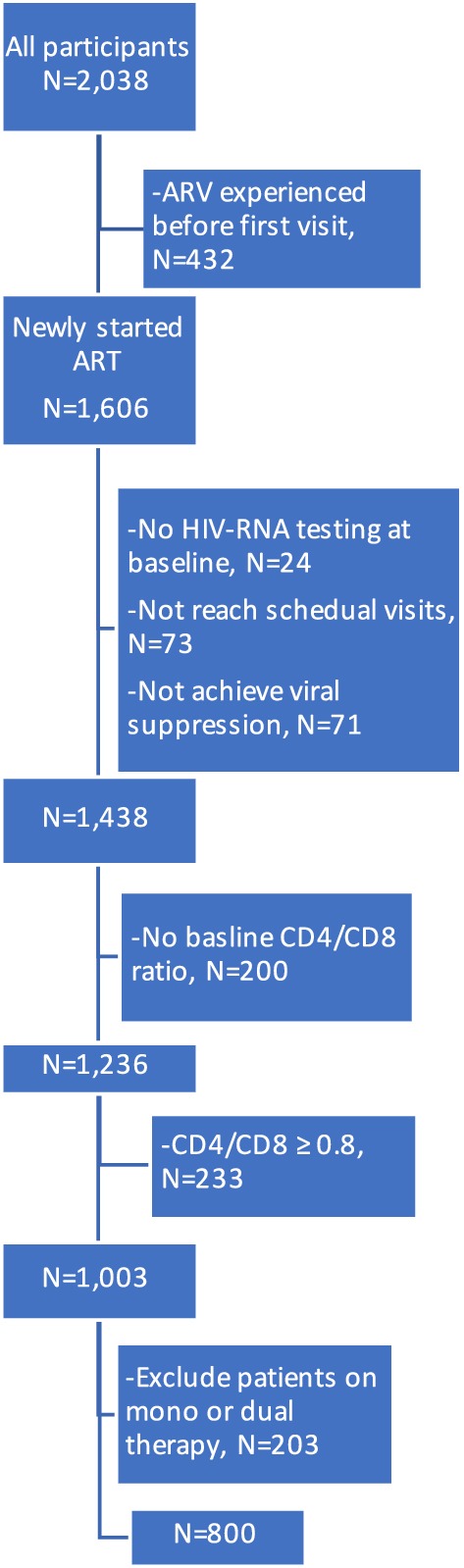
Flow diagram for patients for inclusion to study analysis

After excluding participants treated with cART before the enrollment into the 006 cohort and who did not have baseline CD4/CD8 ratio measurements, 1236 participants were evaluated. We then excluded the patients who had baseline CD4/CD8 ratio ≥ 0.8 and those who commenced on mono or dual ART regimens which may have biased ratio recovery times (Fig. 1). Finally, a total of 800 HIV-infected adults, with 67% male, median age of 32.3 (interquartile range, IQR, 27.4–37.5) years and median baseline HIV-RNA level of 4.8 (IQR, 4.39–5.24) log_10_ copies/mL, achieved viral suppression and were included in the analysis. Of 800 participants, most were heterosexual (54%) and the rest were homosexual or bisexual (35%) injecting drug users (0.8%) and others (11%), with a median duration of 7 (IQR, 4–35) months to achieve the first virological suppression. Median duration of cART over follow-up was 8.9 (IQR, 5.2–13.7) years. Most participants (47%) achieved virological suppression within 6 months and their median duration of suppression was 6.08 (IQR, 2.97–10.75) years (Table 1). Participants who achieved virological suppression within 6 months had higher pre-cART CD4/CD8 ratio (0.23 vs. 0.21, p = 0.04) and lower pre-cART HIV RNA level (4.72 log_10_ vs. 4.85 log_10_ copies/mL, p < 0.005), compared to those achieving virological suppression later. There were no differences in pre-cART CD4, CD8 counts or CDC staging between the two groups. Overall baseline CD4/CD8 ratio before cART initiation was 0.22 (IQR, 0.12–0.32) and median CD4/CD8 ratio at confirmed first time of virological suppression was 0.42 (IRQ: 0.27–0.56).

**Table 1:** Patient characteristics

	N	Descriptive, median (IQR) or n (%)	Not achieved CD4/CD8 ratio < 1 (N = 606)	Achieved CD4/CD8 ratio ≥ 1 (N = 194)	p-value
Gender, n (%)	800				< 0.001
Male		533 (66.63)	439 (72.44)	94 (48.45)	
Female		267 (33.38)	167 (27.56)	100 (51.55)	
Age at start ARV	800	32.3 (27.4–37.5)	31.8 (26.7–37.1)	33.7 (28.6–38.5)	0.006
Pre-cART HIV-RNA level (log_10_ copies/mL)	800	4.8 (4.39–5.24)	4.83 (4.44–5.25)	4.67 (4.18–5.2)	0.003
Pre-cART CD4 count (cells/mm^3^)	799	206 (102–291)	193 (80–282)	229 (170–316)	0.0001
Pre-cART CD8 count (cells/mm^3^)	769	853 (611–1190)	890 (616–1219)	818 (596–1109)	0.16
Pre-ART CD4/CD8 ratio	769	0.22 (0.12–0.32)	0.20 (0.10–0.30)	0.28 (0.18–0.39)	< 0.001
CDC grading, n (%)	790				0.045
A		466 (58.99)	338 (56.71)	128 (65.98)	
B		223 (28.23)	174 (29.19)	49 (25.26)	
C		101 (12.78)	84 (14.09)	17 (8.76)	
Route of transmission, n (%)	800				0.002
Heterosexual		433 (54.13)	305 (50.33)	128 (65.98)	
MSM/bisexual		280 (35)	233 (38.45)	47 (24.23)	
IDU		6 (0.75)	5 (0.83)	1 (0.52)	
Others		4 (0.5)	4 (0.66)	0 (0)	
Unknown		77 (9.63)	59 (9.74)	18 (9.28)	
Months since cART start to first virological suppression		7 (4–35)	7 (3–34)	9 (4–38)	0.51
Duration of cART (years)	800	8.9 (5.2–13.7)	7.0 (4.2–12.1)	13.5 (9.3–16.7)	< 0.001
Duration of viral suppression (years)	800	6.08 (2.97–10.75)	4.91 (2.26–9.12)	10.77 (7.49–12.74)	< 0.001
CD4/CD8 ratio at first viral suppression, median (IQR)	800	0.42 (0.27–0.56)	0.38 (0.24–0.5)	0.54 (0.42–0.69)	< 0.001
CD4/CD8 ratio at first viral suppression, n (%)	800				< 0.001
< 0.3		224 (28.00)	209 (34.49)	15 (7.73)	
0.30–0.45		228 (28.50)	182 (30.03)	46 (23.71)	
> 0.45		348 (43.50)	215 (35.48)	133 (68.56)	
Positive HBsAg, n (%)	784	130/784 (16.58)	108/592 (18.24)	22/192 (11.46)	0.028
Positive anti-HCV Ab, n (%)	796	66/796 (8.29)	50/602 (8.31)	16/194 (8.25)	0.980
Baseline ARV regimen, n (%)	800				< 0.001
PI-based regimen		272 (34)	178 (29.37)	94 (48.45)	
NNRTI-based regimen		509 (63.63)	411 (67.82)	98 (50.52)	
INSTI-based regimen		19 (2.38)	17 (2.81)	2 (1.03)	

Descriptive data are n (%) or median (*IQR* interquartile range), *ART* antiretroviral therapy, *MSM* men who have sex with men, *IDU* injecting drug users, *ARV* antiretroviral drugs, *NNRTI* non-nucleoside reverse transcriptase inhibitor, *PI* protease inhibitor, *INSTI* integrase strand transfer inhibitors

**Fig. 2:**
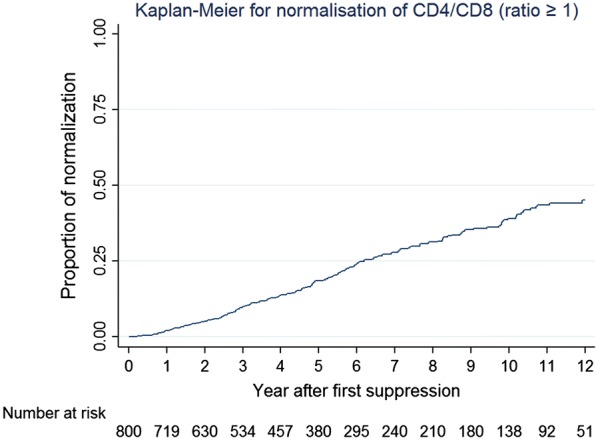
Kaplan–Meier curve showing probabilities of CD4/CD8 ratio normalization

**Fig. 3:**
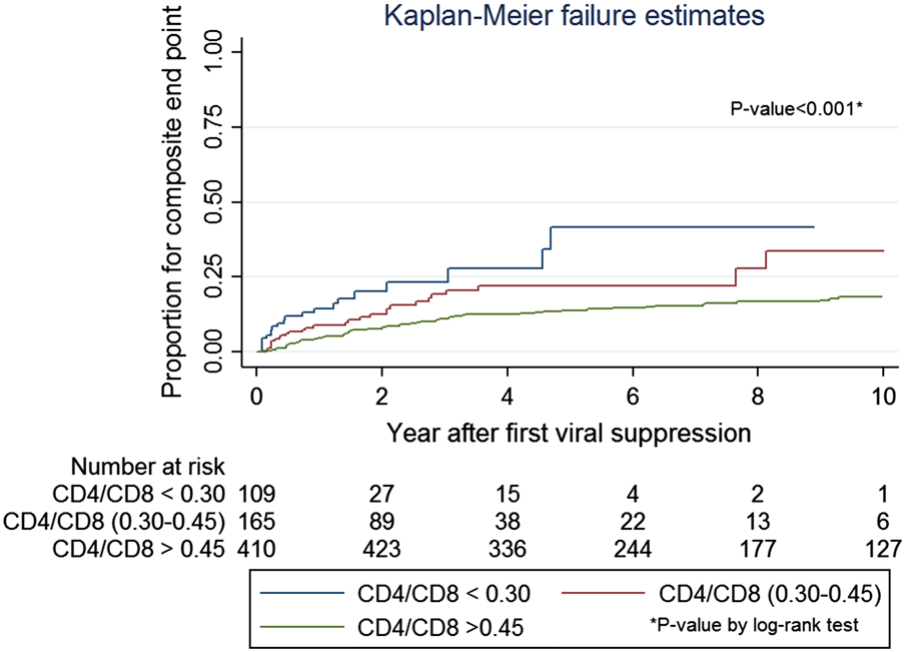
Kaplan–Meier curves showing probabilities of composite end point with CD4/CD8 ratio

The overall incidence rate of CD4/CD8 ratio normalization was 4.38 per 100 PYFU (95% confidence interval [CI] 3.81–5.04) with 4289 PYFU. With Kaplan–Meier estimation methods, we found that the probabilities of normalization at 2, 5 and 10 years after virological suppression were 5.1%, 18.6% and 39.1%, respectively (Figs. 2 and 3). Thirty-six percent of those who achieved normalization had pre-cART CD4 counts ≤ 200 cell counts, compared to 52% of patients who failed to achieve normalization (p < 0.001). Median pre-cART CD4/CD8 ratios for participants achieving normalization and the non-achiever group were 0.20 (IQR, 0.10–0.30) and 0.28 (IQR, 0.18–0.39), respectively. Of note, the duration of virological suppression was longer among the patients who achieved the normalization target than the non-normalization group (10.8 years [IQR: 7.5–12.7] vs. 4.9 years, [IQR: 2.3–9.1], p < 0.001). However, there was no significant difference in the time to first virological suppression between patients who achieved normalization and who did not have normal ratios (p = 0.243). Additionally, there was significant difference in CD4/CD8 ratio at first virological suppression between two groups, with median value of 0.38 (IQR: 0.24–0.5) in patients without CD4/CD8 normalization and 0.54 (0.42–0.69) in the group achieving normalization (p < 0.001).

In multivariate analysis (Table 2), after adjusting for CDC category, months since starting cART to first viological suppression, HBsAg status and baseline cART regimen, factors independently associated with CD4/CD8 ratio normalization were female sex (hazard ratio [HR]: 2.47, 95% CI 1.71–3.56, p < 0.001) and baseline CD4 counts ≥350 cells/mm^3^ (HR: 3.62, 95% CI 2.36–5.55, p < 0.001) or between 200 and 350 cells/mm^3^ (HR: 1.95, 95% CI 1.40–2.72, p < 0.001) vs. < 200 cells/mm^3^ as reference. The interaction between age and CD4/CD8 ratio was tested in the model by using the ratio as continuous, and grouped in categories that matched the analysis. However, no interactions were found.

**Table 2:** Factors associated with normalisation of CD4/CD8 (ratio ≥ 1)

Variables	Univariate			Multivariate		
	HR	95% CI	p-value	HR	95% CI	p-value
Gender						
Male	Ref					
Female	1.99	(1.5–2.64)	*< 0.001*	2.47	(1.71–3.56)	*< 0.001*
Age, (per year older)	1.001	(0.98–1.02)	0.906			
Body mass index (BMI, kg/m^2^)						
< 25	1.11	(0.75–1.64)	0.606			
≥ 25	Ref					
Pre–cART HIV-RNA level (log_10_ copies/mL)	0.79	(0.65–0.96)	0.017			
Pre-cART CD4 count (per 100 cell/mm^3^ increased)	1.40	(1.28–1.54)	*< 0.001*			
Pre-cART CD4 count (cells/mm^3^)			*< 0.001*			*< 0.001*
0–200	Ref			Ref		
201–350	2.05	(1.5–2.81)	*< 0.001*	1.95	(1.4–2.72)	*< 0.001*
≥ 351	3.47	(2.31–5.2)	*< 0.001*	3.62	(2.36–5.55)	*< 0.001*
Pre-cART CD8 count (per 50 cell/mm^3^ increase)	0.99	(0.97–1.004)	0.139			
CDC grading						
A, or B	1.81	(1.1–2.99)	0.019	1.2	(0.71–2.02)	0.503
C	Ref			Ref		
Months since ART start to first virological suppression			0.057			0.25
< 6 months	Ref			Ref		
6–12 months	0.48	(0.22–1.06)	0.07	1.30	(0.84–2.02)	0.24
≥ 12 months	1.14	(0.79–1.64)	0.49	1.49	(0.89–2.51)	0.13
HBsAg status						
Negative	1.62	(1.04–2.52)	0.04	1.26	(0.79–2.01)	0.33
Positive	Ref			Ref		
Anti-HCV Ab status						
Negative	1.08	(0.65–1.81)	0.76			
Positive	Ref					
Baseline ARV regimen			*0.017*			0.14
NNRTI based	Ref					
PI based	1.50	(1.13–1.99)	0.01	1.35	(0.99–1.82)	0.051
INSTI based	2.01	(0.49–8.27)	0.33	1.60	(0.38–6.70)	0.52
cART by calendar year						
< 2007	Ref					
≥ 2007	0.85	(0.61–1.2)	0.37			

*IQR* interquartile range, *ART* antiretroviral therapy, *MSM* men who have sex with men, *IDU* injecting drug users, *ARV* antiretroviral drugs, *NNRTI* non-nucleoside reverse transcriptase inhibitor, *PI* protease inhibitor, *INSTI* integrase strand transfer inhibitors

Significant *p*-values are in italics (*p* < 0.001)

### Non-AIDS defining events

We next explored the associations of CD4/CD8 ratio and NAEs in the second part of our analysis. A total of 123 NAEs occurred in a total of 4071 person-years of follow up. Among them, 108 participants developed chronic kidney disease, 5 developed cardiovascular or cerebrovascular events, 12 developed non-AIDS malignancies and 15 participants died. Among them, 16 patients had more than one of the events mentioned. Overall incidence of NAEs as first events was 3.02 (2.53–3.61) per 100 person-years of follow-up (100 PYS). The incidence rates of non-AIDS composite events were significantly different between the cutoffs for CD4/CD8 ratio, with 11.42 per 100 PYS (95% CI 7.45–17.52) for current CD4/CD8 ratio < 0.3 and 2.23 per 100 PYS (1.77–2.81) for ratio > 0.45 (Table 3).

**Table 3:** Number of composite endpoint events with CD4/CD8 ratio

	No. of event	Person-year of follow-up	Rate per 100 person-year, 95% CI
Composite endpoint (n = 692)	123	4071	3.02 (2.53–3.61)
Current CD4/CD8 ratio*			
< 0.3	21	184	11.42 (7.45–17.52)
0.30–0.45	28	496	5.64 (3.90–8.17)
> 0.45	72	3227	2.23 (1.77–2.81)

* *P* < 0.001 for long rank test

Univariate and multivariate analyses of the risk factors associated with composite endpoint of NAEs are summarized in Table 4. Older age, MSM or bisexual (vs. heterosexual), time updated CD4/CD8 ratio (< 0.3 and 0.3–0.45 vs. > 0.45), diabetes mellitus (DM), baseline protease inhibitors-based regimen and cART initiation after calendar year 2007 (vs. before 2007) were associated with higher chances of non-AIDS composite end point in univariate analysis. However, after adjusting the potential cofounders from the univariate analysis, older age (HR: 1.09, 95% CI 1.05–1.13, p < 0.01) and CD4/CD8 ratio (HR: 3.02, 95% CI 1.27–7.21, p = 0.01) for ratio < 0.3 and between 0.3 and 0.45 (HR: 2.03, 95% CI 1.03–3.98, p = 0.04) vs. > 0.45 were independently associated with higher risks of progression to NAEs in the multivariate analysis. Since the majority of NAE in our cohort were from CKD, the diagnosis of which is derived from an equation, we conducted a sensitivity analysis where CKD was confirmed at 2 consecutive visits. Using this method, 48 patients met criteria for CKD, and we found significantly elevated risks of NAE in multivariate models: HR 3.29 (95% CI 1.66–6.50; p = 0.001) for current CD4/CD8 ratio < 0.3) and HR 2.46 (95% CI 1.44–4.20; p = 0.001) for those with ratios between 0.3 and 0.45, vs. > 0.45 as the reference group.

**Table 4:** Factors associated with composite end point

Variables	Univariate model			Multivariate model		
	HR	95% CI	p-value	HR	95% CI	p-value
Gender						
Male	Ref					
Female	1.09	(0.75–1.58)	0.65			
Age^a^, year	1.07	(1.04–1.09)	< 0.001	1.09	(1.05–1.13)	*< 0.001*
BMI^a^, kg/m^2^						
< 25	Ref			Ref		
≥ 25	1.68	(0.89–3.16)	0.11	1.42	(0.73–2.76)	0.30
Route of transmission			0.008			0.19
Heterosexual	Ref			Ref		
MSM/bisexual	0.54	(0.35–0.82)	0.004	1.74	(0.95–3.21)	0.07
IDU/others/unknown	0.66	(0.33–1.30)	0.23	1.06	(0.32–3.53)	0.93
Ever smoking	0.82	(0.56–1.20)	0.31			
Ever alcohol drinking	0.82	(0.38–1.77)	0.62			
Baseline HIV-RNA level (log_10_ copies/mL)	1.19	(0.92–1.54)	0.19			
Baseline CDC grading						
Stage A or B	Ref					
Stage C	1.03	(0.61–1.74)	0.92			
Tuberculosis infection^a^						
No	Ref					
Yes	1.24	(0.17–8.93)	0.83			
Time since ART initiation to first viral suppression			0.057			
< 6 months	Ref					
6–12 months	0.48	(0.22–1.06)	0.07			
≥ 12 months	1.14	(0.79–1.64)	0.49			
Current CD4 counts per 100 increased	0.93	(0.85–1.02)	0.12			
Current CD4/CD8						
< 0.3	3.10	(1.87–5.14)	< 0.001	3.02	(1.27–7.21)	*0.001*
0.30–0.45	1.76	(1.13–2.75)	0.01	2.03	(1.03–3.98)	*0.04*
> 0.45	Ref			Ref		
Diabetes mellitus	2.27	(1.52–3.39)	< 0.001	3.02	(0.06–163.26)	0.59
HBs Ag status						
Negative	Ref					
Positive	0.69	(0.40–1.18)	0.17			
Anti-HCV Ab status						
Negative	Ref					
Positive	1.04	(0.54–1.98)	0.92			
Baseline ARV regimen						
NNRTI-based	Ref			Ref		
PI-based	1.55	(1.09–2.22)	0.015	1.09	(0.6–1.99)	0.77
cART by calendar year						
< 2007	Ref			Ref		
≥ 2007	0.40	(0.27–0.61)	< 0.001	1.29	(0.7–2.36)	0.42

Significant *p*-values are in italics

*IQR* interquartile range, *BMI* body mass index, *MSM* men who have sex with men, *IDU* injecting drug users, *ARV* antiretroviral drugs, *ART* antiretroviral therapy, *NNRTI* non-nucleoside reverse transcriptase inhibitor, *PI* protease inhibitor, *INSTI* integrase strand transfer inhibitors

^a^Time-updated variables include age, BMI and tuberculosis infections

## Discussion

In our cohort with median duration cART of 8.9 years and median duration of virological suppression of 6.1 years, the probability of achieving CD4/CD8 normalization at 5 and 10 years was 19% and 39% respectively. These Kaplan–Meier probabilities are consistent with our incidence rate of approximately 4% per year. Our data confirmed the previous few available data demonstrating the prolonged duration of recovery to normal CD4/CD8 ratio among PLHIV with suppressive cART, with a median of 10 years to reach normalization target [12]. This study also demonstrated that low ratio was a higher risk for clinical progression of NAEs. This can be translated into that persistent disparity between CD4 and CD8 cell counts, even with CD4 recovery after cART, could be a critical marker for evaluating the prognosis of long-term virologically suppressed PLHIV. Our patients had a relatively lower incidence of CD4/CD8 ratio normalization compared to an Italian cohort. The Italian cohort that included only patients with undetectable HIV viral load (HIV-RNA level < 80 copies/mL) after cART initiation, showed 29% achieved ratio normalization by 5 years whereas it was 19% by 5 years from our study [12]. However, the baseline median CD4/CD8 ratio and median nadir CD4 count in the Italian cohort were higher than those of our Thai cohort (0.39 vs. 0.22, and 378 vs. 206 cells/mm3, respectively). Our study showed that higher chances of normalization were associated with higher baseline CD4 counts (> 350 cells/mm^3^). This is consistent with previous studies suggesting baseline CD4 counts < 200 cells/mm^3^ was an unfavorable predictor towards CD4/CD8 ratio normalization [3, 12], and that low CD4 T-cell counts at baseline before cART are associated with incomplete immune response even after years of viral suppression [20]. Moreover, improvement of CD4/CD8 ratio was seen in both normalization and non-normalization groups after having suppressed viremia, compared to baseline values before cART initiation. This suggests that current ART guidelines recommending initiation of cART treatment with higher CD4 cell counts will have benefits in increasing the number of participants achieved higher CD4/CD8 ratios earlier.

The literature is conflicting regarding the use of CD4/CD8 ratio as a surrogate marker to describe immune dysfunction and to predict the progression towards NAEs and mortality [12, 13, 21]. A report from Mussini et al. [12] suggested the predictive value of CD4/CD8 ratio for clinical progression of severe NAEs and deaths among patients with prolonged cART treatment in a large Italian cohort, with the same cutoff values of the ratio used in our study. In contrast, in a large collaborative study of European and North American cohorts failed to show a link between low CD4/CD8 ratio and non-AIDS related mortality among virologically suppressed patients on cART [21]. Of note, the study showed low ratio and high CD8 counts were associated with AIDS-related mortalities. We sought to carry out an additional analysis to explore whether CD4/CD8 ratio has an association with development of NAEs. Our case definition of NAEs was similar to the ones used in previous studies, which included cardiovascular and cerebrovascular diseases, non-AIDS defining cancers, kidney diseases and deaths [12, 13]. The incidence rates of composite non-AIDS endpoints were significantly different among categories with different CD4/CD8 ratio cutoff values in our study. Patients with low CD4/CD8 ratio had higher incidence rates of NAEs than those with higher ratios. In multivariate analysis, we also found the independent associations of low CD4/CD8 ratio with composite end point or deaths after adjusting for potential confounders that were significant in the univariate analysis.

Previous reports have also suggested the association of CD4/CD8 ratio in T-cell activation or senescence [22, 23]. A plausible explanation associations with NAEs is that low CD4/CD8 ratio could be a marker of ongoing immune dysfunction among prolonged virologically suppressed HIV patients on stable cART since immune activation is been associated with clinical events and mortality in PLHIV [24].

One of the strengths of our study is the prolonged follow-up time with a median of 8.9 (IQR, 5.2–13.7) years of cART treatment. Our participants were taking cART with prolonged duration of virological suppression of 6.1 (IQR, 3–10.8) years. Another strength of our study is that we evaluated the probabilities of normalization among virologically suppressed participants up to 10 years. This is relatively longer than other studies which showed the normalization probabilities just up to 2 years [3] and 5 years [12], respectively.

Several important limitations in this study should be acknowledged. First, we could not provide the association of low CD4/CD8 ratio with each particular non-AIDS incident due to low numbers of events in each category. Second, we could not include some potential factors that have previously suggested may have influences on immune activation or persistent inflammation such as cytomegalovirus (CMV) co-infection [25, 26] in our study analysis. Third, our study population could be considered low risk, since they started cART, and maintained good adherence and virological suppression. Nevertheless, this also allowed us to assess CD4/CD8 ratio recovery without the confounding effect of viremia. Fourth, the study was a retrospective analysis from a prospective cohort. The conclusion for causal inference between low CD4/CD8 ratio and NAEs is therefore low due to the observational nature of the cohort. Lastly, although CD4/CD8 ratios may be a useful marker, there is no known strategy apart from taking cART to improve ratios that are low. Further analyses should explore whether newer therapies for HIV treatment such integrase strand inhibitors or monoclonal antibodies have additional impact on CD4/CD8 ratio recovery.

## Conclusions

Probabilities of immune recovery among patients on cART is relatively low in our cohort, suggesting the ongoing immune dysfunctions even after years of treatment. Patients with low baseline CD4 counts were less likely to achieve ratio normalization. Earlier ART initiation may result in increased normalization rates. We also sought to use multiple NAEs as a composite endpoint to determine its association with the ratio. The finding of low ratio with the linkage to NAEs highlights the potential use of CD4/CD8 ratios as a marker for the clinical progression of non-communicable diseases and mortality among PLHIV with prolonged suppression of viremia.
